# Interviewing Teen Parents: Simulated Patient Experience for Clinical Education and Outreach

**DOI:** 10.7759/cureus.31149

**Published:** 2022-11-06

**Authors:** Peter Averkiou, Lisa C Martinez, Mario Jacomino

**Affiliations:** 1 Women's and Children's Health, Florida Atlantic University Charles E. Schmidt College of Medicine, Boca Raton, USA; 2 Medicine, Florida Atlantic University Charles E. Schmidt College of Medicine, Boca Raton, USA

**Keywords:** outreach programs, underrepresented in medicine, medical school students, simulation in medical education, teenager, preclinical medical students, simulated patients, pediatrics, clinical skills education

## Abstract

Introduction: Many medical students’ initial experience obtaining a history from a pediatric patient happens in their clerkship years. There is a shift in medical education to provide early clinical experiences to train physicians. To increase the exposure to pediatric history in the pre-clinical years, we developed this simulation-based session involving students in their second year of medical school. They are tasked with eliciting a history from a baby provided by a teenager who functions both as a standardized patient (SP) and the parent of the infant. Our goal was to have second-year medical students learn and practice interviewing an adolescent while obtaining history about an infant to assist in the transition to Year three Pediatric clerkship.

Approach: Collaborating with the Office of Diversity at our medical school, we recruited students registered in medical academies in public middle and high schools in our county and asked them to be part of this simulation-based activity. A majority of these medical academy students are underrepresented in medicine (URiM). The students functioned as SPs for pre-clerkship medical students while gaining exposure to a career in medicine and the medical school environment. The medical students obtained a history, with faculty providing formative feedback, followed by documentation of the encounter.

Outcomes: Medical students felt they gained skills to communicate with caregivers of pediatric patients. They also practiced the skill of eliciting a pediatric history from an infant whose parent is a teenager. The middle and high school students that functioned as SPs gained a better appreciation for the medical education system and felt that the experience was valuable for all parties involved.

Discussion: This session exposed pre-clerkship medical students to the nuances of eliciting a pediatric history from pediatric caregivers while also engaging URiM from middle and high school in the medical education process. This session could be used at other institutions to expand diversity in the medical field while also providing pre-clerkship medical students with pediatric experiences.

This article was previously presented as an oral presentation at the AAMC Group on Education Affairs (GEA) Virtual Regional Spring Meeting on April 21, 2021.

## Introduction

Simulation is an active learning approach, and is used in pre-clerkship medical school curricula to teach clinical skills [1]. In addition, simulation supports cognitive integration by providing an explicit, practical application of basic science knowledge in a relevant clinical context [2]. This exposure then serves as a reference point to guide actions that lead to improved patient care delivery and outcomes [3]. During simulation, educators employ standardized patients (SP), people who may or may not be professional actors, and they are instructed on how to act as if they have a particular disease or condition in a health care setting [4]. A recent systematic review suggests that the involvement of children and adolescents in simulation as SP for education and assessment purposes is valuable and feasible [5]. Minors have functioned as SPs in pediatric clinical skills assessments for residents [6] and in third and fourth year of medical school [7], but is less common in the undergraduate medical education curriculum, especially in the pre-clerkship phase.

Pediatrics is a specialized field in medicine, and learners with limited personal experience or exposure to children can find communication, interaction, assessment, and the provision of developmentally appropriate care difficult [8]. The ability to elicit a diagnostically relevant and accurate history is a crucial skill for medical trainees [9]. Medical students are taught clinical skills in their pre-clerkship years of medical school either via modules and videos or on adult patients functioning as SPs [8,10,11]. The few pediatric SP sessions currently published are for clerkship or post-clerkship students [6,7] and other healthcare professional schools [12]. Our medical students attend pre-clerkship preceptor experiences where they see adult patients in their practices. As a result, they get very limited pediatric patient exposure during these real-life experiences. As recommended by other authors, this simulation-based activity was utilized as an adjunct to patient care experiences, and its integration into the curriculum was planned and outcome-driven [13]. In addition, previous groups of second-year medical students requested to include more pediatric topics in their pre-clinical curriculum to help them transition to Pediatric Clerkship in their third year of medical school.

This technical report describes a simulation-based teaching session created for pre-clerkship medical students in the first semester of the second year of medical school to learn how to elicit a pediatric history of a baby where the caregiver is an adolescent SP. These medical students learned how to elicit a complete medical history from an adult during their first year of medical school using adult SP. Eliciting the history from a historian, instead of the patient, is a skill that the pre-clerkship students also must learn. The purpose of our study is to: (1) have second-year medical students obtain a pediatric history, (2) obtain a history from a secondary source, in this case, a parent, and (3) provide exposure to middle and high school students to what happens in medical school.

This article was previously presented as an Oral Presentation at the AAMC Group on Education Affairs (GEA) Virtual Regional Spring Meeting on April 21, 2021.

## Technical report

This simulation session uses middle and high school students as SPs. Recruitment of middle and high school students was done in collaboration with the Office of Diversity at our medical school. This office implements the Community Engagement & Pathway Program at medical academies in middle and high schools in our county. Medical academies in these middle and high schools are designed to provide their students an opportunity to develop the skills needed to compete for, enter, and graduate from health professions schools once they finish high school. Pathway programs have demonstrated increased engagement of high school students from diverse backgrounds in the healthcare professions [14,15]. Similar programs in the US have demonstrated that pathway initiatives that seek to diversify the health professions by providing academic enrichment in the medical sciences and college admissions support to very low-income high school students have been highly successful in reaching low-income students and preparing them for medical and other careers [16]. We approached the members of the Community Engagement and Pathway Program to identify 8 to 10 students from one of the middle or high schools to serve as SPs for our case scenario. The students chosen for this activity were female and proficient in English.

We created an SP scenario (Table 1) to mimic an interaction between the learner and a parent of a one-month-old baby with nasal congestion. We developed the simulated scenario for medical students to gain concrete and in-depth knowledge about a specific real-world situation. Our simulated scenario and the involvement of middle and high school students provide several perspectives. First, it enhances the learning experience of the pre-clerkship medical students by obtaining the medical history of an infant from a historian to learn the differences when eliciting a pediatric medical history as compared to an adult history. Second, it provides an opportunity to obtain a history from a caregiver who is not the patient, in this case, a parent. Third, it presents the opportunity for the medical student to demonstrate empathy, especially when dealing with a common chief concern that is usually benign like nasal congestion, which could be easily dismissed by healthcare professionals. Fourth, it illustrates an interactive way to include middle and high school students in the medical school curriculum and, at the same time, enhance their exposure to a medical school environment while functioning as SPs to possibly increase their interest in pursuing a career in medicine.

**Table 1:** SP case

CASE INFORMATION – Provided by the standardized patient (SP) and only answer if the students asks the specific question.	
Setting the interview	“The name of the baby is John.” You can state your name as the mother or provide another name that you would like to be called.
Establishes patient's comfort	You agree to proceed with the interview and medical history of your baby.
Assesses patient's needs	You have no time constraint at this time and do not need an interpreter.
Initial information & identifying data	“The name of the baby is John.”; “He is one month old.”
Chief Complaint: The patient’s primary reason for seeking care.	“The baby has nasal congestion.”, “I was supposed to return for follow up in a few days, but came today because my baby is having congestion, and I thought that something is wrong with him.”
Additional Concerns: Other concerns the patient has.	“None”
History of Present Illness (HPI):	
The SP will be asked about the attributes of a symptom.	You will answer the following for each attribute:
Onset - (when; from the beginning and until the present time) and has it happened before	“My son has been having some nasal congestion for about a week. This has never happened before.”
Place/Location – where in the body? And does it move/radiates to another place?	“He is having trouble breathing through his nose. It stays there in the nose and doesn’t seem to move anywhere.”
Quality – descriptor of the symptom	“It is noisy breathing.”
Remitting and exacerbating factors – what make the symptom better and/or worse	“I haven’t noticed anything that makes it better or worse. I tried to use a bulb syringe, but that didn’t work.”
Severity – scale of 0 to 10	“It is like a 5 and has to breathe through his mouth.”
Setting - what was going on or where was patient that contribute to the symptom	“It happens at home and my mother’s house. We don’t take John out of the house. It seems worse in the morning.
Timing and Duration – frequency, constant or intermittent	“It’s been pretty constant for the last week, and it happens all day long.
Associated symptoms – other symptoms noted	“I haven’t noticed any rashes or coughing. I noticed clear secretions from the nose for the last week. Nobody at home is sick. He is otherwise acting, eating, sleeping, peeing, and pooping as his usual self.”
Review of systems:	
No other symptoms noted.	No fever, rashes, vomiting, diarrhea, coughing, irritable or fussy.
Past medical history	
Prenatal and birth history	The baby is one month old, and the mother is 16 years old. The baby was born vaginally with no problems. He was born head first and was in labor for about 10 hours. Mother was at home and noticed that water broke after about 8 months of pregnancy. The fluid was clear, and a lot of it came out. The mother of the SP took her to the hospital. The SP’s mother was surprised because she did not know SP was pregnant. Pregnancy was not planned. If female SP: “My periods started when I was 12 years old, and they are regular every 28 days.”; “Because the periods are so regular, I suspected that I was pregnant when I missed one. I went to the pharmacy and bought a pregnancy test, and it was positive.” Mother had a lot of pain during delivery, and it was bad so they gave her some medicine (epidural) in her back (spine = epidural). No illnesses during pregnancy. Pregnancy was hidden because the mother did not gain a lot of weight (about 12 pounds) and wore big shirts/sweaters. No alcohol consumption, tobacco or vaping use, or drug use during pregnancy. No prenatal care/did not go to the doctor during pregnancy. They did STD tests were done during labor and were negative. The baby did not require the use of forceps, CPR, or other medical procedures and was born healthy. I don’t know what an APGAR score is. The baby was discharged two days after birth and was to see the doctor when the baby was two weeks old. Everything went well at that two-week-old visit. The blood type of the baby is unknown. SP has is type B and a positive Rh factor. The birth weight of the baby was 5lbs and 6 oz., and the length 19 inches. The baby is not taking any medications. The baby sleeps well (2-3 hours) and wakes up to eat. He does not seem to be sick except for the congestion.
Childhood Illnesses	None
Surgeries	None
Hospitalizations	Since his discharge from hospital after he was born, John has not been hospitalized.
Ob/Gyn history	Not applicable for this case
Psychiatric history	None
Accidents/ injuries/ trauma	None
Screening tests	John had a hearing test and blood taken from his heel before he was discharged. They were all normal.
Medications	John is not taking any medications.
Allergies	None
Alternative or complementary health care	None
Family history	
Mother, Father, Siblings, Grandparents, and other significant family members.	I don’t know of any illnesses in the family.
Social history	
Birthplace, Marital Status & Living Conditions	The baby was born in Florida at St. Mary’s Hospital one month ago. The mother of the baby is single. The baby lives with the mother and the maternal grandparents of the baby in a house. There are no other adults in the house. John’s mother has one younger sister that also lives in the house, who is 11 years old and likes to help care and play with John. The house has three bedrooms, so the baby sleeps in my room in a crib. No pets in the house. No one smokes in the house. Maternal grandparents and help take care of the baby. An aunt of the mother also lives nearby and helps take care of the baby. Maternal grandmother does not work right now and takes care of the baby when the mother attends school during the weekdays. The father of the baby and his parents also come to visit frequently since they also live close by. At the beginning, all grandparents were not very happy about the pregnancy, but now they accepted the situation and are very supportive.
Relationship and education of father and mother	The father of the baby goes to the same high school as the mother, and this is how they met. The father of the baby lives with his parents and comes to visit the baby frequently. He is 17 years old, and he is supportive. The father and the mother of the baby are in a relationship.
Diet (describe)	The baby is fed formula and no breast milk. The baby takes 3 to 4 ounces of formula every three hours. We wake the baby up every three hours to feed him. We get WIC (Women, Infants and Children) vouchers, so which helps us cover the cost of the formula.
Exercise (describe)	None
Educational and employment history	The baby stays with maternal grandmother at home during the day while the mom goes to school.
Religious background/believes	None
Personal habits	The baby is eating, sleeping, peeing and pooping as his usual self.
Activities of Daily Living	Baby does not roll over yet, but does move head towards loud noises.
Significant life events	None
Sexual history	None
Travel history	None
List any other important social history or information important to this case	Mother has been under stress. She returned to school, so John has been staying at home with the maternal grandmother during the day. It has been hard to adjust because of the school work and the baby waking up at night because he is hungry and wants to be fed.
*Physical exam:* The physical exam of the baby or the SP will not be performed in this session.	
*Differential diagnosis:* For faculty to discuss with the students during the debrief section.	

Prior to organizing this learning activity, we submitted and received approval from our University’s Institutional Review Board. Parental consent and child assent were sought with training as per project protocol. As part of our planning process, the parent(s) of the middle or high school students received a copy of the SP scenario prior to the activity for them to review it and provide consent for their child to participate as an SP. We had two sets of parents who declined to consent due to concerns of sexual history. Once parental consent and child assent were obtained, the SPs received a copy of the scenario and initial training from their school teacher. On the day of the simulation, the students arrived at our institution for additional training. Female students played the role of the SPs in our activity, but the scenario could be converted to having a male parent as the historian. Each SP was asked to carry a baby mannequin wrapped in a blanket to mimic a real-world situation.

We planned and completed the activity in two afternoons, with half of the class of second-year medical students attending each session. We reviewed the checklist (Table 2) that was provided to the medical students that highlighted the items to be asked of a pediatric patient and their caregivers. They were able to refer to that checklist during the interaction with the SP once inside the exam room. We utilized eight exam rooms, each with one SP. Two medical students were assigned to each room. A faculty member was present in every exam room to observe the interaction of the medical students with the SP.

**Table 2:** Checklist

Setting the interview: Professionalism	Yes	No
1. Asks permission to enter the room		
2. Greeting and introduction		
3. Establishes patient's comfort		
4. Assesses patient's needs		
Initial Information & Identifying Data	Yes	No
5. Initial information		
6. Identifying data		
Chief Complaint (CC)	Yes	No
7. Elicits Chief Complaint		
8. Obtains patient concerns, sets agenda		
History of Present Illness (HPI)	Yes	No
9. Onset and has it happened before?		
10. Place or location & radiation of symptom		
11. Quality of symptom		
12. Remitting and exacerbating factors		
13. Severity or quantity		
14. Setting		
15. Timing		
16. Associated symptoms		
17. Summary		
Past Medical and Surgical History	Yes	No
18. Prenatal history		
a. age of the mother of the patient		
b. gravida, para, abortions		
c. blood type of mother and child		
d. was the pregnancy planned		
e. prenatal care including location and number of visits		
f. illnesses and complications during pregnancy		
g. special tests, ultrasound exams, stress tests during pregnancy		
d. STDs and viral infections, tests dates and results of mother		
g. medications during pregnancy, prescribed & over the counter		
h. smoking, use of drugs or alcohol during pregnancy		
19. Birth history		
a. spontaneous or induced labor		
b. complications of labor (if any)		
c. fetal monitoring and fetal distress		
d. rupture of membranes including time, spontaneous or artificial		
e. medications during labor and delivery		
f. vaginal or C-section delivery		
g. fetal presentation and position		
h. use of forceps or other equipment		
i. Apgar scores at 1 minute and 5 minutes		
j. resuscitation used, state type and indication (if any)		
k. place of birth (e.g., hospital, home, birthing center)		
l. birth weight and length at birth of the baby		
m. feedings (e.g., breast milk, formula, amount and frequency)		
n. medications given to the baby at or after birth, or currently taking?		
20. Childhood Illnesses		
21. Surgeries		
22. Hospitalizations		
23. Obstetric/Gynecologic History (if applicable)		
24. Psychiatric History		
25. Accidents and Injuries		
26. Immunizations		
27. Screening tests		
28. Medications		
29. Allergies		
30. Alternative/Complementary health care		
Family History	Yes	No
31. Family History		
32. Review Specific Diseases		
Social History	Yes	No
33. Birthplace, Marital Status & Living Conditions		
34. Relationship and education of father and mother		
35. Nutritional and Exercise History		
36. Educational and Employment History		
37. Religious background/belief system		
38. Personal habits		
39. Activities of Daily Living (ADLs)		
40. Significant life events		
41. Sexual history		
42 Travel history		
Review of Systems (ROS)	Yes	No

Inputs and processes

In order to complete this activity, we needed the following resources:

*Physical Space: *Simulation center with eight standardized patient exam rooms, eight infant mannequins, lecture hall

*Documents: *Checklist of benchmarks, SP story, lecture PowerPoint reviewing the pediatric history checklist, “chart” documents for the students to review in advance

*Staff and Faculty*: Staff to recruit and train SPs, partnerships with community public schools, SPs from local middle and high schools, eight faculty to supervise each SP room, one faculty to present the lecture and debrief the session, staff member to run the activity and movements of students and faculty

Pre-briefing

A 30-minute pre-brief session was organized on the day of the session before the interaction with the SP with an MS PowerPoint (Redmond, USA) presentation for the second-year medical students to learn the difference in eliciting a pediatric history and to review the questions in the checklist that the student will be using with the SPs in the exam room. During this time, medical students and faculty were made aware that SPs were adolescent minors with parental consent for the interview process but will not perform a physical exam on the mother or the baby in this simulated case. In addition, each medical student was provided with a “chart” document that has written instructions (Table 3) before entering the exam room to provide previous medical information about the baby, objectives for the activity and instructions as to how to proceed once inside the exam room were also given. 

**Table 3:** Chart document with objectives and instructions for students

Chief complaint: Nasal Congestion
*Age:* One month weight two weeks ago: 6 lbs 15 oz Birth weight: 5 lbs 6 oz Length two weeks ago: 19.75 inches Birth length: 19 inches Head Circumference two weeks ago: 38 cm Head circumference at birth: 36.5 cm
*Objectives:* By the end of the session, the student should be able to: Identify the differences between eliciting the medical history in pediatric patients as compared to adult patients. Obtain a medical history of an infant from a guardian (teen parent) professionally and respectfully. Incorporate feedback from faculty, peers, and standardized patients to create personal learning/improvement goals for the future.
*Instructions:* You will be taking a history from a mother who reports that her baby has nasal congestion. The mother, who is the standardized patient, is a middle or high school student. You can use the Pediatric history benchmarks to proceed with the medical history. No physical exam will be conducted in this session.

Case

The simulation-based activity involves a teen mom who brings her baby to be seen at the outpatient pediatric clinic. Two medical students were assigned to enter each exam room to interview the SP who provided the medical history of their baby in the role of a parent/caregiver. Thirty minutes were allotted for the medical student to complete the interview with the SP inside the exam room. The students were given this extended amount of time because it was the first time they had elicited a history from someone other than the patient and their first time obtaining a pediatric history. Instructions were given for one student to elicit the chief complaint, history of present illness, and review of systems, and the second student to elicit the remainder of the history, including past medical history, prenatal history, and social and family history. A faculty member was present in every exam room to observe the interaction of the medical students with the SP. The faculty avoided interrupting their interaction for the first 30 minutes and provided formative feedback to medical students in the last 15 minutes of the activity inside the exam room. The students’ performance was assessed with a formative evaluation by the faculty using the Yes or No checkboxes on the checklist. The SP did not provide feedback to the medical students. After 45 minutes, all medical students and faculty returned to the main lecture room to debrief the activity and answer any questions.

Debrief

In debrief, we established a safe and confidential space for students and faculty to present their experiences during the simulation-based activity. The debrief process aimed to solicit the experience of the medical students when interacting with an adolescent, eliciting information from a caregiver, and the differences in obtaining a medical history in a pediatric experience compared to an adult. These debrief also allowed the identification of the impact of the experience, gaps in knowledge, and process errors.

Outcomes

The second-year medical students that participated in the activity completed an evaluation of eight questions utilizing a 5-point Likert scale and one qualitative question for students to write additional feedback. To evaluate the experience of the SPs, one of the authors met with all SPs after the completion of each session to debrief. As mentioned in the literature, group debriefing may benefit adolescents [17]. Their teacher also accompanied the SPs during this period. A group of 64 pre-clerkship medical students completed the activity. Fifty-one students (80%) completed the evaluation form. The activity was well received, as noted in Table 4.

**Table 4:** Average scores for each question completed by students in post-session evaluation. (N=51) Rated on a 5-point scale: (1) Strongly Disagree; (2) Disagree; (3) Neutral; (4) Agree; (5) Strongly Agree

Rate the extent that:	Average score
The objectives of the clinical skills session were clear.	4.30
The clinical skill session helped me refine my history taking skills.	4.22
The introduction to the pediatric history was relevant to my medical education.	4.20
The time allotted to the clinical skills encounter was sufficient.	4.16
The online resources were helpful for this session.	4.08
The clinical skills faculty provided helpful feedback.	4.42
The structure and organization of the session was appropriate for practicing history taking from a pediatric patient.	4.00
This session has improved my understanding of the pediatric history.	4.00

The qualitative question provided positive responses, including: “I learned how to take the history of a newborn pediatric patient via the mother or guardian. I also learned to make sure to focus on the newborn instead of the mother as the patient.” “It was good to have middle school students be our SPs for today's session because it gave some perspective in terms of the expected medical literacy and also the experience of talking to someone younger.”

The meeting with the SPs at the end of the activity resulted in three main topics:

1. They enjoyed the field trip to the College of Medicine and exposure to the facility, being an SP, the learning environment that mimics a medical office, and the interaction with the medical students, staff, and faculty.

2. They agreed that training medical students on how to elicit a history of a pediatric patient are imperative and that the use of SPs as a vehicle to accomplish that goal makes it engaging and memorable before meeting a real adolescent in the clinical years.

3. They realized that pregnancy and parenting as a teenager can create a significant lifestyle change.

Middle and high school students were used as caregiver SPs to enhance the training of medical students in the pre-clerkship years. The benefits to the middle and high school students that functioned as SPs included learning outside the standard classroom environment and exposure to a medical school environment, which could lead to an interest in pursuing a career in the healthcare professions. We included middle and high school students from underrepresented groups in public schools in our simulation-based activity, but students from different backgrounds or other schools (private or charter schools) could be asked to be the SPs.

## Discussion

We began this study wanting to prepare a simulation-based activity with the goal of (1) having students obtain a pediatric history, (2) obtaining a history from a secondary source, in this case, a parent, and (3) providing exposure to middle and high school students to what happens in medical school. As demonstrated in our results, students found this session to be helpful to their understanding of pediatric history and felt that the experience was relevant to their medical education. They also felt the format and use of middle and high school students helped meet the learning objectives. The SPs also felt that the session was worthwhile. However, there were some limitations to the session:

(1) Parental consent. A larger group of middle and high school students must be available to account for the situation when parents may not consent to the script;

(2) As middle and high school students are not professional SPs, it requires a relatively simple story/scenario to which they can relate easily;

(3) The physical exam was not performed in this activity to avoid issues with examining minors;

(4) Coordination of the schedule of the middle and high school students to fit the schedule of the medical school students, as we only held the activity on two specific afternoons. The dates need to be reserved as early as possible during the planning process;

(5) There are many resources necessary, including space and faculty time. Other institutions may have more exam rooms available and can accommodate more students per session and limit the activity to only one afternoon to streamline any scheduling conflict.

## Conclusions

This technical report submission is significant for two reasons. First, pre-clerkship medical students learned to elicit a pediatric history from a teenage SP who is the caregiver of her infant. Second, it utilized middle and high school students as caregiver SPs. These two concepts are gaps in the literature. More importantly, we achieved our main goal for pre-clerkship medical students to learn the differences when eliciting the medical history in pediatric patients, as compared to adults, before entering their clerkship years. This simulated activity was prepared for pre-clerkship medical students to elicit the medical history in 30 minutes. It can be adapted in a shorter amount of time in the case of more experienced students. The activity was scheduled over two afternoons for educational purposes but also can be a stand-alone formative or summative assessment or included as a station in an Objective Structured Clinical Exam (OSCE) format. The checklist was used consistently by faculty for formative assessment of the medical students. It could also be used to provide a summative assessment to students following the OSCE format.
